# Supplementary material for: Comparative genomics provides new insights into the diversity, physiology, and sexuality of the only industrially exploited tremellomycete: Phaffia rhodozyma
Source: BMC Genomics. 2016 Nov 9;17:901. doi: 10.1186/s12864-016-3244-7 (PMC5103461; doi:10.1186/s12864-016-3244-7)
Supplement: Additional file 6: — List of orphan genes with links to PFAM (related to Additional file 1: Table S1). (ZIP 1428 kb) [file 12864_2016_3244_MOESM6_ESM.zip › PFAM_UNIQUE/G03621_P.html]

#                                                                            --- full sequence --- -------------- this domain -------------   hmm coord   ali coord   env coord
  
# target name        accession   tlen query name           accession   qlen   E-value  score  bias   #  of  c-Evalue  i-Evalue  score  bias  from    to  from    to  from    to  acc description of target
  
#------------------- ---------- ----- -------------------- ---------- ----- --------- ------ ----- --- --- --------- --------- ------ ----- ----- ----- ----- ----- ----- ----- ---- ---------------------
  
G03621\_P             -            287 HPIH                 PF13323.1    153     0.053   11.9   0.0   1   1   1.9e-05      0.11   10.8   0.0    57   139    78   167    64   175 0.86 - N-terminal domain with HPIH motif  
G03621\_P             -            287 PD40                 PF07676.7     39     0.041   12.3   0.6   1   1   0.00024      0.15   10.5   0.6    16    30   113   127   113   128 0.97 - WD40-like Beta Propeller Repeat
